# Distortion Correction and Denoising of Light Sheet Fluorescence Images

**DOI:** 10.3390/s24072053

**Published:** 2024-03-23

**Authors:** Adrien Julia, Rabah Iguernaissi, François J. Michel, Valéry Matarazzo, Djamal Merad

**Affiliations:** 1 LIS, CNRS, Laboratoire d’Informatique et des Systèmes, Centre National de la Recherche Scientifique, Aix Marseille University, 13284 Marseille, France; 2 INMED, INSERM, Institut de Neurobiologie de la Méditerranée, Institut National de la Santé et de la Recherche Médicale, Aix Marseille University, 13284 Marseille, France; francois.michel@inserm.fr (F.J.M.);

**Keywords:** light sheet fluorescence microscopy, neuroscience, preprossessing, denoising, deconvolution, axial distortion, deep learning, auto-encoder

## Abstract

Light Sheet Fluorescence Microscopy (LSFM) has emerged as a valuable tool for neurobiologists, enabling the rapid and high-quality volumetric imaging of mice brains. However, inherent artifacts and distortions introduced during the imaging process necessitate careful enhancement of LSFM images for optimal 3D reconstructions. This work aims to correct images slice by slice before reconstructing 3D volumes. Our approach involves a three-step process: firstly, the implementation of a deblurring algorithm using the work of K. Becker; secondly, an automatic contrast enhancement; and thirdly, the development of a convolutional denoising auto-encoder featuring skip connections to effectively address noise introduced by contrast enhancement, particularly excelling in handling mixed Poisson–Gaussian noise. Additionally, we tackle the challenge of axial distortion in LSFM by introducing an approach based on an auto-encoder trained on bead calibration images. The proposed pipeline demonstrates a complete solution, presenting promising results that surpass existing methods in denoising LSFM images. These advancements hold potential to significantly improve the interpretation of biological data.

## 1. Introduction

In fluorescence microscopy, a sample is exposed to an illumination beam. Part of this radiation is absorbed by the sample, and the other is released through fluorescence. Fluorescence is a mechanism where a fluorophore absorbs a photon and emits another photon a few nanoseconds later, with lower energy than the incident photon [1]. This loss of energy induces a longer wavelength for the emitted photon than the absorbed photon, and this shift is called the Stokes shift. It is also possible to absorb two photons at the same time and emit a single higher-energy photon. This phenomenon, called two-photon fluorescence microscopy, allows us to use a lower-energy light as an excitation source. As a result, light with longer wavelengths can penetrate tissues to a greater depth. Also, lower-energy light induces less damage to observed cells.

While imaging a sample through fluorescence microscopy, the emitted light must be kept and the excitation light rejected. This separation is performed by using optical filters and considering that a fluorophore with a broad Stokes shift will result in a less contaminated image. Choosing a filter and an indicator always involves a compromise; the overlap between excitation and emission wavelengths can produce spectral bleed (or cross talk), which affects the quality of the resulting image. This cross talk may lead to increased noise in the image.

This paper considers the case of widefield fluorescence microscopy, where the whole sample is illuminated at the same time. The resulting emitted light is collected through an objective lens and then focused onto a camera with a tube lens. Illuminating the whole sample enables fast imaging compared to other methods. While fast imaging a sample, the excitation intensity is reduced, which avoids photobleaching and phototoxicity. Conversely, confocal microscopy uses a different scheme: a laser beam excites a small part of the specimen, requiring a scanning of the whole specimen to form an image.

However, widefield microscopy suffers from reduced resolution if the fluorescent objects are too close together. The fluorescent emitted light follows Airy rings due to diffraction, which can overlap. Overlapping enfolds the objects in a blurry spot where the Rayleigh criterion is not satisfied.

The ability of widefield microscopy to image a wide region in a short time allows us to perform 3D imaging of biological samples, which is a powerful tool for neuroscience. A particular case of widefield microscopes, Light Sheet Fluorescence Microscopy (LSFM), can generate high-quality images at various scales. In contrast to other imaging techniques, it achieves optical sectioning by employing a plane of illumination rather than laser point scanning. The sample is illuminated from the sides by a thin light sheet, and the fluorescence emission is captured along the axis perpendicular to the illumination plane. A camera is directed toward this plane, capturing photons emitted by the fluorescent sample. By adjusting the plane of illumination (or the sample itself) along the detection axis, a 3D volume of the sample can be reconstructed. This approach ensures minimal phototoxicity and offers rapid volumetric imaging compared to confocal or two-photon microscopes while maintaining excellent contrast [2]. For the widespread application of light sheet microscopy to extensive biological tissue, it is necessary to integrate clearing protocols that render the specimen transparent. This is commonly accomplished by replacing water with a medium possessing a refractive index that falls between that of proteins and lipids.

The microscope used in this study is the Ultramicroscope from LaVision Biotec (Bielefeld, Germany). This microscope is a Light Sheet Fluorescence Microscope, using a sheet of light measuring between 4 and 10 µm thickness. To minimize artifacts, the light sheet is segmented into three sub-beams converging toward the sample at varying angles. This approach helps prevent the occurrence of shading artifacts commonly associated with this technique. The illumination can be obtained from the left, the right, or both sides, depending on the sample imaged.

The sensor used on the camera of this microscope is a CMOS having a resolution of 2560×2160 pixels and a bit depth of 16 bit. The objectives mounted provide magnifications from 1.26× to 12.6×. The excitation source is a pulsed picosecond white laser coupled to a spectrally dispersing fiber generating a supercontinuum ranging from 450 nm to more than 900 nm. As a result, objects around 1cm^3^ could be imaged at a µm resolution on the x−y plane and at 2 µm on the *z* axis.

In this study, we explored various image-processing approaches to enhance the quality of Light Sheet Fluorescence Microscopy (LSFM) images. We propose an extended version of a study previously published in conference proceedings [3,4]. Our focus encompassed multiple aspects of these images, beginning with the removal of the instrumental response, namely the Point Spread Function (PSF), a well-studied aspect in microscopy. However, eliminating the PSF often amplifies noise. To address this, we introduced a denoising auto-encoder (DAE) to mitigate the resulting noise, comparing it with traditional denoising methods and evaluating the effectiveness of deep learning approaches.

Additionally, we aimed to enhance contrast, particularly in regions such as filaments, to further improve the overall image quality. Finally, we proposed a deep learning solution to rectify distortions along the z-stack in LSFM images. In microscopy, liquid immersion objectives are commonplace, with an increased refractive index enhancing the numerical aperture of the objective lens and consequently improving resolution. However, when imaging cleared samples of the brain immersed in a specific medium (such as DBE or water in our experiments), a refractive index mismatch between the sample and the objective immersion medium can occur.

**Figure 1:**
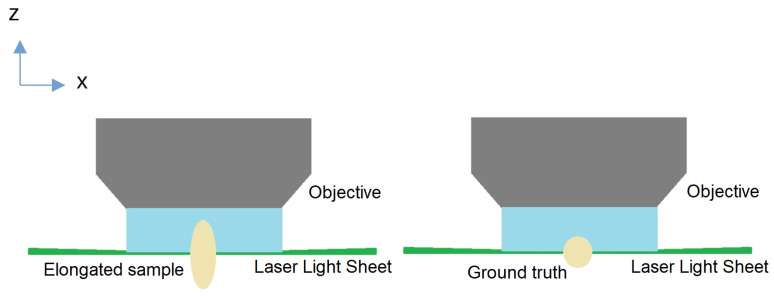
Distortion along the z-stack on a Light Sheet Fluorescent Microscope.

This mismatch introduces several undesirable effects, particularly during the reconstruction of the entire 3D volume. Spherical aberration leads to a loss of the axial resolution, resulting in compression or elongation along the depth axis, as illustrated in Figure 1. To address this issue, our proposed method employs an auto-encoder to learn the inverse transform, thereby correcting distortions in 3D stacks.

The proposed paper first investigates the existing methods aiming to correct LSFM images from degradations. Then, a slice-by-slice pipeline is developed by using a deblurring and a contrast-enhancement algorithm. A denoising auto-encoder was constructed for the last step of the pipeline, obtaining better results than the existing literature on LSFM images. Lastly, we developed a novel approach for correcting image stacks from axial distortion encountered in LSFM. As previously discussed in [3,4], this study extends the findings presented in our earlier conference paper.

## 2. Related Work

Improving the quality of LSFM (Light Sheet Fluorescence Microscopy) images has been an actively researched area in recent years. Various approaches have been proposed to tackle this challenge. The proposed works focused on either deblurring or removing artifacts. Deblurring is a critical task in processing images captured by optical devices, where the recorded image results from the convolution of the observed image with the Point Spread Function (PSF) of the system, leading to a degradation in the observation of fine details. To address this issue, two suitable approaches emerge: measuring the PSF (using fluorescent beads) or estimating it through blind deconvolution. Modeling the PSF for fluorescence images presents challenges because the system’s PSF is a combination of the objective PSF and the fluorescence illumination PSF. The limited width of the observed PSF, determined by the objective and camera resolution, complicates accurate measurement. For instance, when employing a wide-field objective (2× or 4×), the observed PSF is only a few pixels wide. Consequently, many approaches dealing with this challenge rely on iterative models to estimate the PSF effectively.

Among these studies, refs. [5,6] employ an iterative model utilizing the Richardson–Lucy algorithm to estimate the PSF. In this process, the resulting image is a combination of the restored image convolved with the PSF and some Poisson noise. The iterative algorithm aims to determine the optimal estimate of the restored image, matching it with the recorded image convolved by the PSF. A quality criterion is defined to gauge the difference between each restored image during iterations. In their more recent publication [6], an additional preprocessing step is introduced, involving a 3D rolling ball filter. The radius is set to the minimum size of structures of interest. This preprocessing step effectively eliminates contaminated background signals before the deconvolution process, preventing the amplification of such signals.

Another method based on the Richardson–Lucy algorithm was proposed by [7], dealing with the 3D deconvolution of multiview light sheet microscope images. They consider a spatially varying PSF model, calibrated by using irregularly placed fluorescent beads.

The Richardson–Lucy deconvolution method is not the only iterative approach for estimating a restored image. In [8], a Kalman-based minimum variance linear estimation algorithm is proposed with the dual objectives of noise reduction and deblurring in microscopy images. This method relies on a minimal set of parameters, avoiding the generation of artifacts, and is computationally efficient.

For thick light sheet images obtained by using different optical setups, ref. [9] presents a comprehensive pipeline. It involves the removal of Poisson–Gaussian mixed noise slice by slice (by using a side window filtering technique), deconvolution of the 3D image stack, and elimination of dark stripe artifacts introduced by their deconvolution scheme. Their PSF model is constrained by using fluorescent beads (alternatively, a simulated PSF generated by a PSF Generator [10] could be employed). The deconvolution algorithm is regularized with a Hessian term to maintain continuity in the image stack.

The work of [11] focused on the relatively understudied z-axis elongation (or compression) aberration encountered in light sheet microscopy. This aberration, arising from the refractive index (RI) mismatch between the immersion medium and the sample, is canceled by computing an axial distortion-correction factor. This correction factor is then applied during the reconstruction of the 3D model.

Another study explores the scenario of spatially varying deconvolution. In [12], an image-formation model was developed to simulate the physics of a light sheet microscope, incorporating both the objective Point Spread Function and the light sheet illumination. The optical aberrations within the pupil function of the objective PSF are fitted with a linear combination of Zernike polynomials and constrained through a least square regression of bead images. A variational model is then defined, taking into account this image-formation model and a mixed Poisson–Gaussian noise model. The optimization is carried out through a total variation regularization term and a fidelity term.

Optical strategies tailored for LSFM have been developed to address its challenges. In [13], a modified light sheet microscope, iSPIM, was introduced as one such approach. Departing from the conventional use of a Gaussian beam for illumination, they implemented a 0th-order Bessel beam. Bessel beams exhibit propagation without succumbing to the effects of diffraction. The side lobes introduced by the Bessel beam create an out-of-focus background, which can be eliminated through complementary beam subtraction (CBS). The noise and blurring resulting from the CBS imaging are corrected by using a compressed blind deconvolution and denoising algorithm (CBDD). Instead of using this algorithm, requiring double scanning and heavy computations, they developed an alternative deep learning method called CBS-Deep. Their method significantly enhances the PSNR and resolution of Bessel-beam-based light sheet images.

An alternative optics-based strategy was designed, as presented by [14]. In their research, they created an optical module capable of detecting and compensating for aberrations. The wavefront is reconstructed by measuring the spinning-pupil aberrations. These measurements control a deformable lens, which corrects the aberrations in the center of the field of view. Similar to adaptive optics, the system operates in a closed loop, continuously refining corrections. The residual aberrations on the edges are finally corrected by using anisoplanatic deconvolution.

Other approaches aiming to correct light sheet images from degradation have been proposed. Ref. [15] developed a method for removing stripe artifacts stemming from absorbing or scattering structures present in microscope images. Their approach lies in the use of deep learning through a UNet-based auto-encoder with residual blocks and attention modules. This subject has also been extensively studied in the review [16], which compiles various optical solutions, post-processing strategies, and hybrid approaches aimed at addressing this particular issue.

An alternative method to enhance image quality involves background subtraction. When reconstructing a 3D model from z-stack images, the background noise can envelop the region of interest in a blurred pattern, especially when using Maximum Intensity Projection. In [17], a highly effective approach for eliminating this background signal was introduced through a virtual HiLo based on edge detection (V-HiLo-ED). In contrast to standard HiLo imaging, which demands specific hardware setups, their method achieves background-free image reconstruction by using only a single-slice image.

Ref. [18] developed a U-Net-based network to restore fluorescence microscopy images from low light conditions. Their method lies in a network trained on pairs of high- and low-exposure images, allowing one to restore images from noise and blur. An increase in the axial resolution is also observed by using the CARE algorithm. However, the PSF is supposed to be constant inside the whole imaged volume, and their method needs to be trained on specific biological organisms.

The work of [19] introduced a novel denoising tool known as Noise2void. Their approach focuses on learning a denoising transform without relying on pairs of noise-free and noisy images. A very similar approach has been proposed, Noise2Noise [20], where they used two images of the same scene without ground truth to train their network, providing outstanding results. While their method is designed for efficiency across various image types, we advocate for the use of a specialized auto-encoder tailored for denoising LSFM images. This would enable a more precise fitting to the unique noise profile of LSFM.

The work of [21] provides a very deep convolutional auto-encoder model called RED-Net, using mirroring convolution and deconvolution layers. Each convolution layer is linked by using a skip connection to the corresponding deconvolution layer. Their algorithm proposes to deal with several image-reconstruction modalities, including denoising and non-blind image deblurring. RED-Net includes multiple network depths, going through 10, 20, and 30 convolution layers (with and without skip connections), allowing one to probe the gain obtained by using deeper networks. This approach is in fact quite similar to the one used in this paper regarding the noise reduction part; the difference lies in the fact that we want to build a model specific to LSFM data rather than a general denoising model.

## 3. Materials and Methods

While Light Sheet Fluorescence Microscopy is a potent tool offering cell-specific resolution, images produced by using this technique are not without limitations, including noise and distortion. In this study, we present a comprehensive pipeline designed to address these issues.

Our proposed approach begins by enhancing the contrast of the region of interest (cells) through the removal of the impulse response of the optical system, allowing for better visualization of intricate details. However, the deconvolution and contrast-enhancement processes may introduce some level of noise, prompting the inclusion of a denoising step in our pipeline. We apply these correction steps to each slice image, and once the entire stack is processed, we proceed to correct distortions along the *z*-axis in the 3D reconstructed object.

It is noteworthy that each process is carried out slice by slice in this paper, with 3D reconstruction serving as the final step. This sequential approach is adopted to accommodate the dual nature of biological analysis, conducted in both 3D and 2D dimensions. Consequently, maintaining clear 2D images is imperative for a comprehensive understanding of the biological structures. The strength of our approach lies in the fact that each stage of the pipeline is completely independent, making it possible to easily bypass a correction if it tends to degrade the signal more than it corrects it.

### 3.1. Deblurring

The first step in our method lies in correcting the effects of the system’s impulse response on images. Removing the impulse response (i.e., the PSF) from images allows one to eliminate the blur introduced by the optics. For this purpose, we adopted the approach proposed in [5,6] stipulating that measuring a sufficiently resolved PSF is very difficult with LSFM, thus encouraging one to simulate it. In fact, according to Rayleigh’s criterion and the Nyquist theorem, the camera resolution should be at least 50 megapixels to obtain a resolved PSF (using a 4× objective). This limits the ability to properly measure a PSF of the system.

In this approach, the PSF is derived from an optical model of the image formation of LSFM, allowing one to obtain a resolved PSF at any magnification. Estimating the restored image *O* from the recorded image *D* with a transfer function *H* (the PSF) and some additive noise is expressed as

(1) D=O⊛H+N

Going into Fourier space gives

(2) F(D)=F(O)×F(H)+F(N)

Without noise, the observed image *O* could be retrieved through

(3) $$O=F^{-1}\left(\frac{F(D)}{F(H)}\right)$$

Nevertheless, ref. [5] notices that in practical applications, the direct division by F(H) poses a considerable risk of significantly increasing the additive noise within the image. In turn, this results in a pronounced elevation of high-frequency components toward infinity, especially in regions where F(H) have values close to zero. They used the Richardson–Lucy algorithm to solve this problem. This algorithm considers the recording image as a combination of the true image convolved with the PSF of the microscope plus Poisson noise. Modeling the noise profile with a Poisson model is coherent with the use of CMOS cameras. The RL algorithm is cast as

(4) $$O^{n+1}=O^{\left(n\right)}\left[\frac{D}{H⊛O^{\left(n\right)}}\right]⊛\hat{H}$$

The Richardson–Lucy algorithm is known for its slow convergence and could strongly amplify the noise present in images. The literature addressed this issue by using either Tikhonov–Miller regularization or total variation regularization. In their paper, ref. [5] used a simpler model called Flux-Preserving regularization, where at each iteration a smoothed version of $O^{\left(n\right)}$ is computed by convolving $O^{\left(n\right)}$ with an average filter. This smoothed version, denoted $O^{*(n)}$, is weighted with a coefficient. Thus, the algorithm follows for each iteration of this equation:

(5) $$O^{n+1}=\left(1-\gamma \right)O^{\left(n\right)}\left[\frac{D}{H⊛O^{\left(n\right)}}\right]⊛\hat{H}+\gamma RO^{\left(n\right)}$$

where γ is the regularization factor and R is the average filter.

To enhance the contrast, we opted for the straightforward Contrast-Limited Adaptive Histogram Equalization (CLAHE) method, as outlined by Zuiderveld in 1994 [22]. This method avoids noise amplification while concurrently improving the Peak Signal-to-Noise Ratio (PSNR). While various other methods have been devised for contrast enhancement, we deliberately selected CLAHE as it represents the gold standard, consistently yielding superior results compared to traditional histogram equalization.

The preference for CLAHE arises from its adaptive enhancement of local contrast, effectively preventing overamplification and clipping, thereby preserving intricate image details. This is particularly advantageous in scenarios with diverse illumination conditions or contrast levels across different regions, such as in fluorescence images.

### 3.2. Denoising

The second step in our pipeline concerns noise reduction. Noise reduction has been widely studied with the development of digital image processing. When dealing with fluorescence images, denoising is even more important: the fluorescence signal emitted by the sample labeled with Green Fluorescent Protein is weak.

Fluorescence microscopy is susceptible to various sources of noise, which can be effectively represented by a Poisson–Gaussian noise model. This model involves a Poisson component that captures shot noise and a Gaussian additive noise model that accounts for thermal noise or other signal-independent contributions. Accordingly, we could express the observed signal $z_{i}$ as the combination of the true signal $y_{i}$ corrupted by noise $n_{i}$, where $n_{g}$ is the Gaussian additive noise and $n_{p}$ is the Poisson component:

(6) $$z_{i}=y_{i}+n_{i}=n_{p}\left(y_{i}\right)+n_{g}$$

The denoising task could be summarized as estimating the signal $y_{i}$ knowing the noisy signal $z_{i}$.

**Figure 2:**
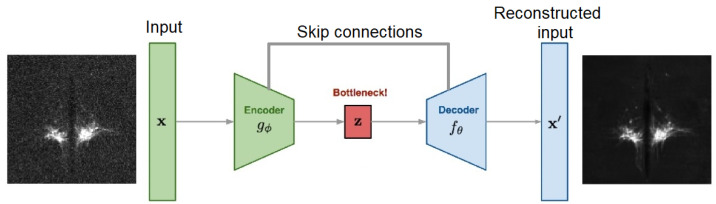
Denoising auto-encoder principle.

Auto-encoders are able to capture a useful representation of an input signal, robust enough for both Poisson and Gaussian noise. Thus, this kind of network is powerful in denoising tasks. Based on the work performed in [21,23], we proposed to use an auto-encoder made of three convolutional layers on the encoder part and three transposed convolutional layers on the decoder part. The network is explained in Figure 2, and the architecture is the following:

**Encoder:**

Input: $128\times 128\times 1\to C_{2D}\left(256,5,5\right)\to BN\to MP_{2D}\to C_{2D}\left(512,3,3\right)\to SD_{2D}\to \;MP_{2D}\to C_{2D}\left(1024,3,3\right)\to BN\to MP_{2D}$

**Decoder:**

$C_{2D}\left(1024,3,3\right)\to BN\to US_{2D}\to C_{2D}^{T}\left(512,3,3\right)\to BN\to C_{2D}\left(256,3,3\right)\to BN\to \;US_{2D}\to C_{2D}\left(1,5,5\right)\to \mathrm{Output}:128\times 128\times 1$

Using the following notations:

- $C_{2D}=$ Conv 2D.
- $C_{2D}^{T}=$ Conv 2D transposed.
- BN= batch normalization.
- $MP_{2D}=$ max pooling 2D.
- $SD_{2D}=$ spatial dropout 2D.
- $US_{2D}=$ up sampling 2D.

We regularized our network by using a spatial dropout layer after the second convolutional layer and a batch normalization layer after each convolutional layer, preventing overfitting. Spatial dropout drops entire 2D feature maps whereas dropout drops individual elements, helping regularize the training if adjacent pixels are strongly correlated. Batch normalization stabilizes and accelerates the training of neural networks by normalizing the input to each layer, reducing internal covariate shifts and sensitivity to hyperparameters.

We added a skip connection between the second convolutional layer of the encoder and the second convolutional layer of the decoder. Skip connections prevent the vanishing gradient problem during backpropagation and assist the decoder in reconstructing a clean image by efficiently passing details forward. We did not observe any edge effects attributable to the convolutions, rendering the use of overlapping patches unnecessary.

The denoising auto-encoder was trained by using a learning rate of $10^{-6}$ through an MSE loss function, the optimization algorithm used was Adam, and the training was conducted on 200 epochs with a batch size of 8.

### 3.3. 3D Distortion Correction

Using an LSFM, we could reconstruct a 3D image at a very high speed and resolution. Nevertheless, optical aberrations limit the quality of the reconstructions. Refractive index heterogeneities between the cleared sample and immersion medium cause light scattering. Light scattering compresses the 3D image if the immersion medium refractive index is lower than the sample refractive index (air) or stretches the 3D image if the immersion medium refractive index is higher than the sample refractive index (oil). Ref. [11] investigated the sources of these aberrations and proposed to compute a correction factor to remove this distortion. This method does not take into account the variation in the distortion inside the x−y−z volume. Correcting this distortion on the z-axis is poorly studied in the literature. For this purpose, we propose, in this work, a more robust method to correct these aberrations on the stacks of images obtained by LSFM.

**Figure 3:**
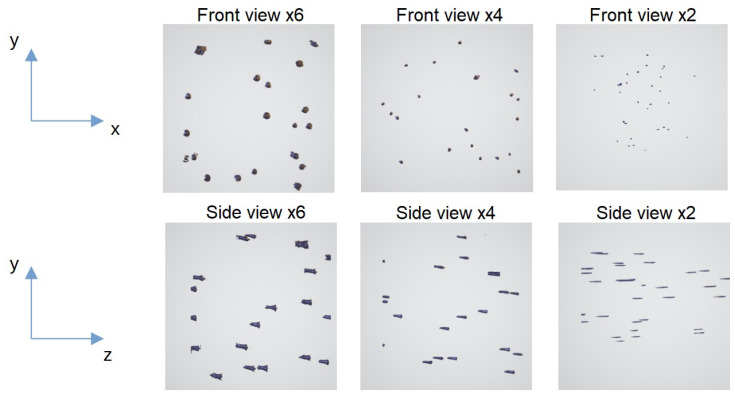
Fluorescent beads reconstructed in 3D by using ImageJ 2.15.0, imaged by using the LSFM at several magnifications.

We used an auto-encoder to learn the inverse transform aiming to correct images from axial distortion. To do so, we determined the distortion by imaging fluorescent beads, which are perfectly spherical. The beads (4 µm diameter) were immersed inside a cube of agarose gel and were imaged with the LSFM at various magnifications (2×, 4×, and 6×). As shown in Figure 3, the elongation along the z-axis is strongly dependent on the magnification, going from a factor 1.8 at 6× magnification to roughly a factor of 10 at 2× magnification.

**Figure 4:**
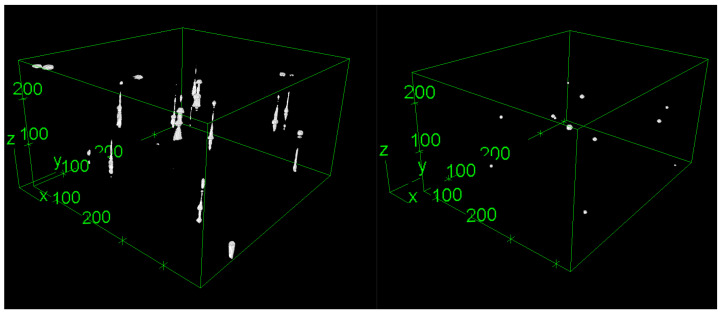
Training dataset: original distorted beads (**left**) and target-corrected beads (**right**), reconstructed by using the 3D viewer from Fiji 2.15.0 [24], pixels units.

We began by generating a training dataset by using these bead images. From the bead image stack, we reconstructed a corrected, non-distorted image stack. Beads were segmented on each slice image by using K-Means clustering. We calculated the slice corresponding to the center of each bead and then reconstructed each bead at the detected position, corrected from elongation. We applied a Gaussian light profile to simulate a grayscale illumination. Ultimately, a virtual bead image stack without distortion mirrors the original distorted image stack, as presented in Figure 4.

**Figure 5:**
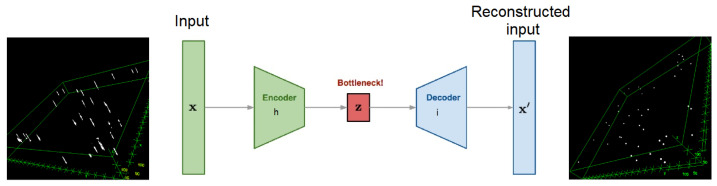
Distortion correction auto-encoder by using convolutional LSTM layers.

Using this dataset as the target dataset, and the original bead image stack as the input dataset, we trained an auto-encoder to learn the transform from the distorted stack to the corrected stack. Figure 5 shows the 3D reconstruction of the input and output datasets.

The auto-encoder is designed by using only two layers, a convolutional layer with 1024 filters of size (3,3) for the encoder and a transposed convolutional layer for the decoder.

The network is optimized through RMSprop and trained by using the binary cross entropy loss function. The LSFM images are 2048×2048; thus, we need to split them into patches in order to feed the auto-encoder without overloading the GPU memory. A patch size of 128×128 is a good choice for keeping away artifacts while reconstructing the corrected images.

LSFM generates stacks of 2D images that can be reconstructed in 3D by using techniques such as Maximum Intensity Projection (MIP). Employing a 2D convolution network for operations along the *z*-axis is not an appropriate choice. Therefore, it is necessary to explore architectures that can handle all three axes. Various options are available in the literature, and we have opted to utilize Long Short-Term Memory (LSTM) auto-encoders. These networks are designed to process sequences of images, originally developed to handle videos (hence, time sequences). A sequence of 31 images is enough to capture the whole distortion on bead images, and this implies the use of patch images of 32×32 pixels. The architecture of the network used this time is the following:

**Encoder:**

Input: $31\times 32\times 32\times 1\to C_{LSTM2D}\left(16,3,3\right)\to C_{LSTM2D}\left(32,3,3\right)$

**Decoder:**

$C_{LSTM2D}\left(32,3,3\right)\to C_{LSTM2D}\left(16,3,3\right)\to C_{3D}\left(1,3,3,3\right)\to \mathrm{Output}:31\times 32\times 32\times 1$

Using the following notations:

- $C_{LSTM2D}=$ Conv LSTM 2D.
- $C_{3D}=$ Conv 3D.

Handling 3D data for training purposes proves computationally demanding compared to 2D data. In our approach, we deliberately opted for a conservative choice of using a limited number of filters to accommodate the computational resources of a standard computer with an 8 GB GPU RAM. However, a more in-depth investigation, potentially utilizing a supercomputer infrastructure, holds the potential to explore the utilization of deeper neural networks with an increased filter capacity for enhanced model performance. We trained this network by using an MSE loss function and Adam optimizer by using a learning rate of $10^{-4}$ and a batch size of 8.

## 4. Results and Discussion

We conducted the assessment of our image-enhancement pipeline in two main parts: the 2D processing stage (deconvolution, contrast enhancement, and denoising) and the 3D corrections.

### 4.1. 2D Image Processing

We began by evaluating the 2D processing steps performed for deblurring LSFM images and removing artifacts. To evaluate our denoising auto-encoder model, we used a dataset of artificially corrupted images with mixed Poisson–Gaussian noise.

This dataset contains 1237 images, among which 1000 were used to train the model and the others for testing. Expanding the dataset is unnecessary; the existing variations in noise are sufficient to address LSFM images. The images utilized in this part, for the evaluation of 2D processing techniques, consist of mouse brain images depicting Hypothalamic OXT-GFP neurons at P0, obtained through the clarified brain CUBIC method. These image stacks exhibit sufficiently low levels of noise, facilitating the incorporation of a noise model for subsequent removal utilizing our algorithm. Then, we compared our results to commonly used approaches based on the PSNR and the SSIM metrics (Table 1).

**Figure 6:**
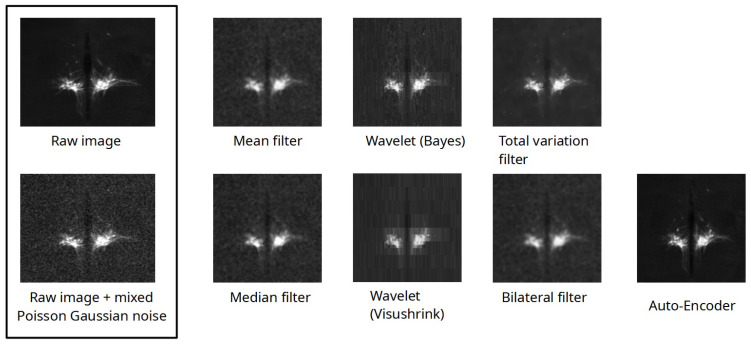
Denoising algorithms compared to an image of Hypothalamic OXT-GFP neurons using P0-clarified brain CUBIC method.

Employing denoising auto-encoders results in a significant improvement in both the PSNR and overall image quality. This enhancement is achieved without introducing any noticeable artifacts, as evidenced by comparisons with other denoising techniques such as wavelet denoising (aliasing) or the smoothing effects observed on the median filter, mean filter, and bilateral filter (refer to Figure 6). The superiority of DAEs in preserving image fidelity while effectively reducing noise becomes apparent through a visual assessment of these comparative results. The total variation provides sharp images while reducing noise, but the overall gain in the PSNR is poor compared to DAEs. Our model performs better than the DAE proposed by [23], providing a higher PSNR and a higher SSIM. The use of skip connections, spatial dropout, and batch normalization reduces the reconstruction artifacts and provides a better PSNR than the architecture submitted by [23].

**Figure 7:**
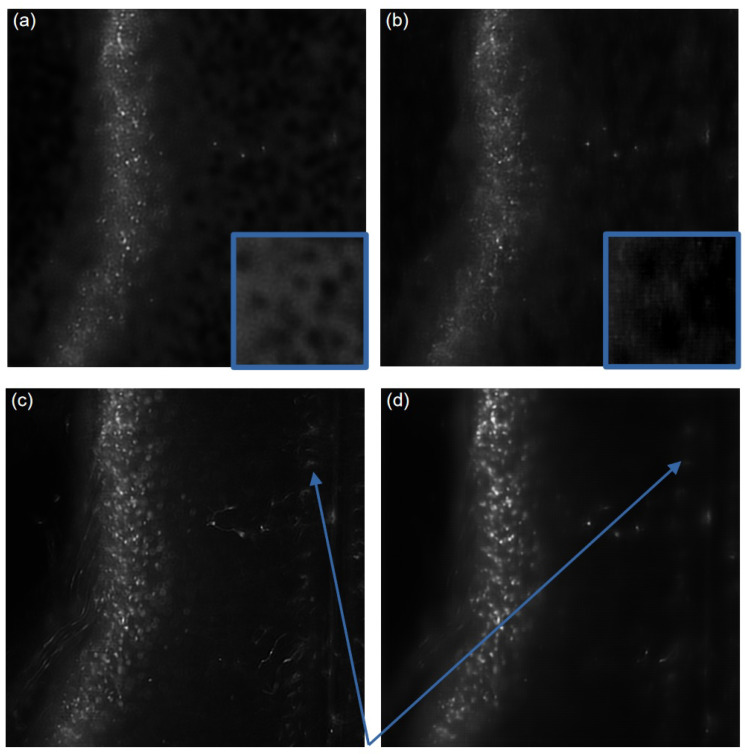
(**a**) DAE from [23], (**b**) our DAE model, (**c**) deblurred image using [5,6], (**d**) full pipeline: deblurring + CLAHE + denoising. The full pipeline is able to increase the contrast of the region of interest while reducing the background signal, as pointed by the arrows.

These artifacts are visible in Figure 7a; the model from [23] induces some dark bubbles on the background while our model is able to reconstruct a strongly corrupted image without introducing such artifacts. The dark bubbles can be observed in the blue insets, which offers a magnified view of a segment of the background in each image (coming from the region behind the insets). The cells are also less blurred by using our model (Figure 7b).

These types of auto-encoders are characterized by their ease and speed of training, particularly on a computer equipped with a decent GPU. Once the auto-encoder is trained, the inference or prediction process is highly efficient and demands minimal computational resources, making it well-suited for applications with constraints on time and hardware resources.

Then, to evaluate the whole 2D-image-processing pipeline based on deblurring LSFM images and removing artifacts, we used the deconvolution algorithm proposed in [5,6], a contrast enhancement (CLAHE), and our DAE model.

**Figure 8:**
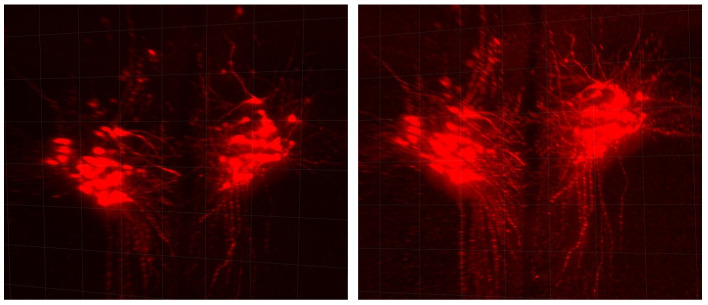
Hypothalamic OXT-GFP neurons obtained by using P0-clarified brain CUBIC method (zoom 4×). Raw stack (**left**), post-processed stack (**right**), front view, reconstructed by using Imaris 9.5, Oxford Instruments (Abingdon-on-Thames, United-Kingdom).

The results of Table 2 and Figure 7 clearly show that applying our full pipeline considerably enhances the quality of LSFM images. In fact, Table 2 indicates a clear increase in the PSNR by using the deconvolution algorithm, the CLAHE algorithm, and the denoising auto-encoder. The combination of these three steps allows for a greater PSNR than each separate step, except for the deblurring, which gives a similar result as the entire pipeline regarding the PSNR. Image quality is subjective; thus, we asked biologists their opinion about our procedure, and it is clear that we gained image quality by using our full pipeline rather than only the deblurring. The full pipeline is able to increase the contrast of the region of interest while reducing the background signal. Specifically, out-of-focus contaminating signals visible in Figure 7c have been completely eliminated in the image shown in Figure 7d. These signals are pointed at by the blue arrows. It is worth noting that eliminating background structures is particularly crucial when reconstructing images into a 3D volume. The synergistic impact of combining the three processing steps becomes evident in Figure 8. Our comprehensive pipeline results in a substantial expansion of visible regions, highlighting a significant improvement in the overall image quality. Importantly, this enhancement is achieved without the introduction of background noise, underscoring the effectiveness of our integrated approach.

### 4.2. 3D Distortion Correction

**Figure 9:**
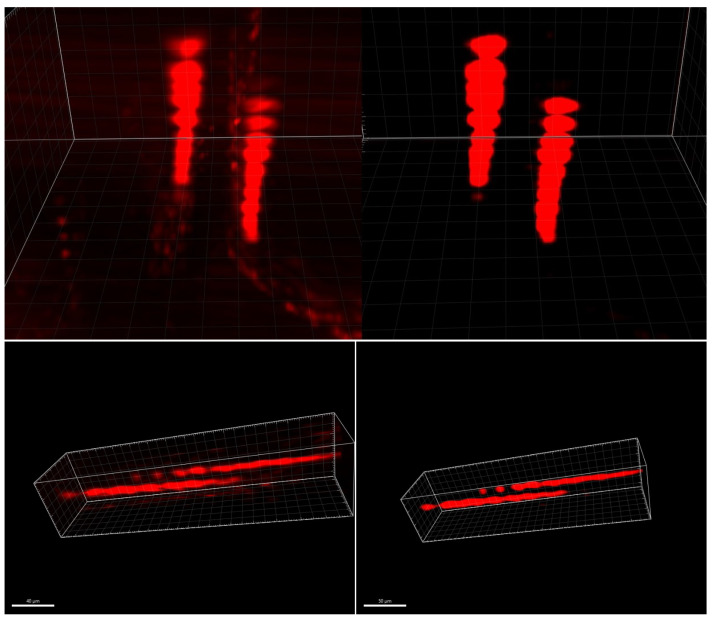
Two neurons, original 3D stack (**left**) and distortion-corrected stack (**right**), reconstructed by using Imaris 9.5, Oxford Instruments (Abingdon-on-Thames, United-Kingdom). The two neurons are elongated along the z-axis, vertically on the top, and horizontally on the bottom.

We obtained initial findings regarding the distortion-correction algorithm by initially applying a two-layer auto-encoder (excluding LSTM components). The results, shown in Figure 9, illustrate the model’s ability to reconstruct 3D volumes without introducing unwanted artifacts that were not present in the original image stack. In the collaborative context with biologists, it is crucial to avoid introducing misleading information into images or 3D models.

These preliminary findings reveal a subtle influence on axial distortion, primarily stemming from the constrained size of our training set. The prototype development involved the use of a downsampled dataset with dimensions of 128×128×280, placing restrictions on the auto-encoder’s effectiveness at capturing the elongation of beads along the *z*-axis. The choice of this subsampled dataset further prevents a direct comparison of our outcomes with the results presented by [11]. Their model is constructed based on simulating the physics inherent to Light Sheet Fluorescence Microscopy and is therefore not tailored for operation with subsampled image stacks. The limited training data size and the downsampling approach contribute to the nuanced impact observed on axial distortion in our initial assessments.

The training process for this network presents a certain level of complexity due to the existence of multiple black patches resulting from the limited size of the beads. Consequently, the auto-encoder exhibits a tendency to average the input signal, resulting in gray images. To address this challenge, a strategic approach involves selecting only those patches where beads are present during training. Additionally, incorporating a network architecture that accounts for the *z*-axis, such as Long Short-Term Memory, is anticipated to contribute to achieving superior results in mitigating the undesired averaging effect and enhancing the overall performance of the auto-encoder in preserving fine details during image reconstruction.

In response to this challenge, we worked on obtaining preliminary results through the application of a two-layer deep LSTM network. Our choice of the training database remained consistent with the one employed in the previous approach. However, in this iteration, we strategically refined our approach by confining the patches exclusively to regions where beads were present, effectively minimizing the occurrence of undesirable black patches. This modification was particularly critical to avoid potential distortions, like the gray results observed before, introduced during the training process.

Furthermore, to accommodate GPU memory constraints, we found it necessary to reduce the size of the patches. This adjustment was imperative for maintaining computational efficiency during the training of the LSTM. It is noteworthy that the unique nature of LSTM training involves considering sequences of images rather than individual frames. This distinctive characteristic prompted careful adjustments in the patch size to optimize the utilization of GPU memory and ensure the seamless processing of image sequences.

**Figure 10:**
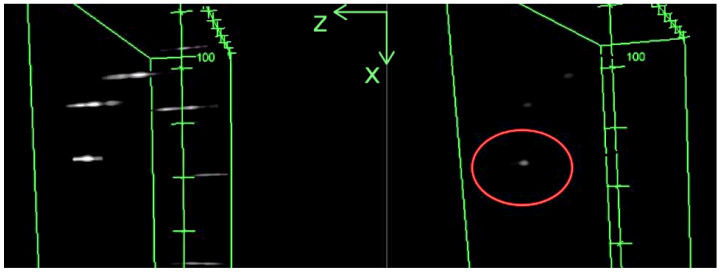
Original 3D stack of fluorescent beads (**left**) and distortion-corrected stack (**right**), reconstructed by using the 3D viewer from Fiji [24]. The z-axis extends horizontally from right to left and is graduated in pixels. The red circle highlights the reconstructed bead.

As illustrated in Figure 10, our latest result using a two-layer deep LSTM network revealed a discernible impact on the elongation phenomenon. However, it is noteworthy that this achievement was made under time and data constraints, limiting the depth of our investigation into the obtained results. Despite this notable progress, a significant loss of contrast was observed in the reconstructed images, posing a challenge to the interpretation of the reconstructed 3D volume.

## 5. Conclusions

The LSFM is able to perform high-quality images of biological samples at different scales. We developed post-processing methods that strongly enhance the information contained in these images.

Our proposed pipeline for correcting slice-by-slice images is able to increase the signal on the regions of interest while reducing the background noise. The deblurring algorithm submitted by [5,6] combined with automatic contrast enhancement increases the ability to distinguish tiny details on biological samples. Our suggested DAE outperforms the results obtained by [23], while also preventing the generation of background artifacts, resulting in efficient background subtraction. Removing background noise is even more important while dealing with 3D images reconstructed through Maximum Intensity Projection (MIP): a low background signal results in embedding the region of interest in a blurred shell.

We addressed the axial distortion encountered in LSFM by using a novel approach based on a convolutional auto-encoder. This method is very easy to implement in a biology lab as it only requires bead image stacks. We obtained preliminary results by using a two-layer deep LSTM network instead of a simple auto-encoder. Given the constraints in our current exploration, we acknowledge the necessity for a more comprehensive investigation into the architecture of the LSTM employed. A more in-depth study of the LSTM architecture holds the promise of uncovering potential refinements that could address the observed contrast issues and contribute to achieving superior results in the distortion-correction process. Additionally, a more extensive examination of our approach is warranted to facilitate quantitative assessments, enabling direct comparisons with the methodology proposed by [11]. Such a comparative analysis would provide insights into the effectiveness of our method in addressing non-uniform distortions, a task that surpasses the capabilities of a simplistic correction factor method. The quantitative evaluation promises to elucidate the nuanced strengths and potential areas of improvement for our proposed distortion-correction methodology.

## Figures and Tables

**Table 1:** PSNR and SSIM comparing conventional methods, the DAE from [23], and our model.

	PSNR (dB)	SSIM
Original + noise	17.123±0.028	0.164±0.001
Bilateral filter	21.541±0.021	0.373±0.001
Median filter	23.410±0.043	0.486±0.002
Total variation	25.324±0.076	0.801±0.003
Wavelet (Bayes)	25.538±0.090	0.807±0.004
Wavelet (Visushrink)	25.654±0.094	0.820±0.004
Mean filter	26.096±0.091	0.841±0.004
BM3D	26.884±0.121	0.886±0.004
DAE (Gondara)	38.564±0.174	0.977±0.001
**DAE (ours)**	39.319±0.191	0.979±0.001

**Table 2:** PSNR evaluation after deblurring, CLAHE, and denoising.

	PSNR (dB)
Deblurring	33.863±5.115
CLAHE	31.609±4.071
Deblurring + CLAHE	29.238±3.835
Deblurring + CLAHE + denoising	33.463±4.108

## Data Availability

Data are available upon request from the corresponding author, and will be provided on a public repository post-publication.

## Abbreviations

The following abbreviations are used in this manuscript:

LSFM	Light Sheet Fluorescence Microscopy
PSF	Point Spread Function
DAE	Denoising auto-encoder
DBE	Dibasic ester
RI	Refractive index
iSPIM	Inverted Selective Plane Illumination Microscopy
CBS	Complementary Beam Subtraction
CBDD	Compressed blind deconvolution and denoising
PSNR	Peak Signal-to-Noise Ratio
SSIM	Structural Similarity Index Measure
RL algorithm	Richardson–Lucy algorithm
CLAHE	Contrast-Limited Adaptive Histogram Equalization
MSE	Mean Squared Error
GPU	Graphics Processing Unit
BM3D	Block-matching and 3D filtering
LSTM	Long Short-Term Memory
